# An accessible, interactive GenePattern Notebook for analysis and exploration of single-cell transcriptomic data

**DOI:** 10.12688/f1000research.15830.2

**Published:** 2019-05-29

**Authors:** Clarence K. Mah, Alexander T. Wenzel, Edwin F. Juarez, Thorin Tabor, Michael M. Reich, Jill P. Mesirov

**Affiliations:** 1 Department of Medicine, University of California, San Diego, La Jolla, CA, 92093, USA; 2 Moores Cancer Center, University of California, San Diego, La Jolla, CA, 92093, USA

**Keywords:** scRNA-seq, single-cell expression, pre-processing, clustering, interactive, visualization, GenePattern Notebook, Jupyter Notebook, open-source

## Abstract

Single-cell RNA sequencing (scRNA-seq) has emerged as a popular method to profile gene expression at the resolution of individual cells. While there have been methods and software specifically developed to analyze scRNA-seq data, they are most accessible to users who program. We have created a scRNA-seq clustering analysis GenePattern Notebook that provides an interactive, easy-to-use interface for data analysis and exploration of scRNA-Seq data, without the need to write or view any code. The notebook provides a standard scRNA-seq analysis workflow for pre-processing data, identification of sub-populations of cells by clustering, and exploration of biomarkers to characterize heterogeneous cell populations and delineate cell types.

## Introduction

Single-cell RNA sequencing (scRNA-seq) is a powerful tool to measure genome-wide gene expression at the resolution of individual cells. Compared to traditional RNA-seq collected from bulk cells or tissue, scRNA-seq enables users to capture cell-by-cell transcriptomic variability. This information can then be used to define and characterize heterogeneity within a population of cells, from identifying known cell types to discovering novel ones. A number of high-throughput scRNA-seq protocols have been developed to simultaneously sequence thousands to hundreds of thousands of cells while retaining the origin of each transcript, including SMART-seq2 ( Picelli *et al*., 2014), CEL-seq ( Hashimshony *et al*., 2012), Drop-seq ( Macosko *et al*., 2015), and the commercial 10X Genomics scRNA-seq protocol. Despite the power of this approach, analysis of scRNA-seq data presents a unique set of challenges centered on the discrimination of technical variation from the biological signal. The variability in efficiency of capturing individual transcripts is compounded by the variability in the number of transcripts per cell, anywhere between 50,000 to 300,000 ( Marinov *et al*., 2014). Conversely, reads for multiple cells may be captured together, artificially inflating the number of reads for a single cell. Comprehensive methods and software have been developed for proper data pre-processing, normalization, quality control, and clustering analysis including Seurat ( Satija *et al*., 2015), Scanpy ( Wolf *et al*., 2018), and the 10X Genomics Cell Ranger pipeline. These methods take raw read counts as input and are downstream of read alignment and quantification. They have been used successfully in studies across many cell types to analyze tens of thousands of cells in parallel ( Macosko *et al*., 2015; Svensson *et al*., 2018; Villani *et al*., 2017).

While these tools are readily available for those with computational expertise who are comfortable programming in Python or R, they are less accessible to non-coding users due to a steep learning curve. In order to enable analysis of scRNA-seq data, regardless of programming expertise, we have created an interactive analysis notebook using the GenePattern Notebook Environment that does not require coding by the user ( Reich *et al*., 2017). The GenePattern Notebook Environment integrates an easy-to-use graphical user interface with the Jupyter notebook's rich text, media, executable code, and results, to present the entire narrative in a single notebook document.

The notebook presented here aims to provide a standard pre-processing and clustering analysis workflow for scRNA-seq datasets. We based the workflow on the Seurat R tutorial and perform the below analysis steps using methods implemented in the Scanpy Python package.

## Methods

### Setup analysis

The workflow begins with an expression data matrix already derived from alignment of reads and quantification of RNA transcripts. Users may upload a single expression file and specify whether the rows represent genes and the columns represent cells or vice-versa. Text files from read count quantification tools like HTSeq ( Anders *et al*., 2015) and Kallisto ( Bray *et al*., 2016) are supported as input. Additionally, this notebook supports the three-file 10X output format, allowing users to upload the matrix, genes, and barcodes files. Any of those inputs can also be provided as .zip files.

**Figure 1.**
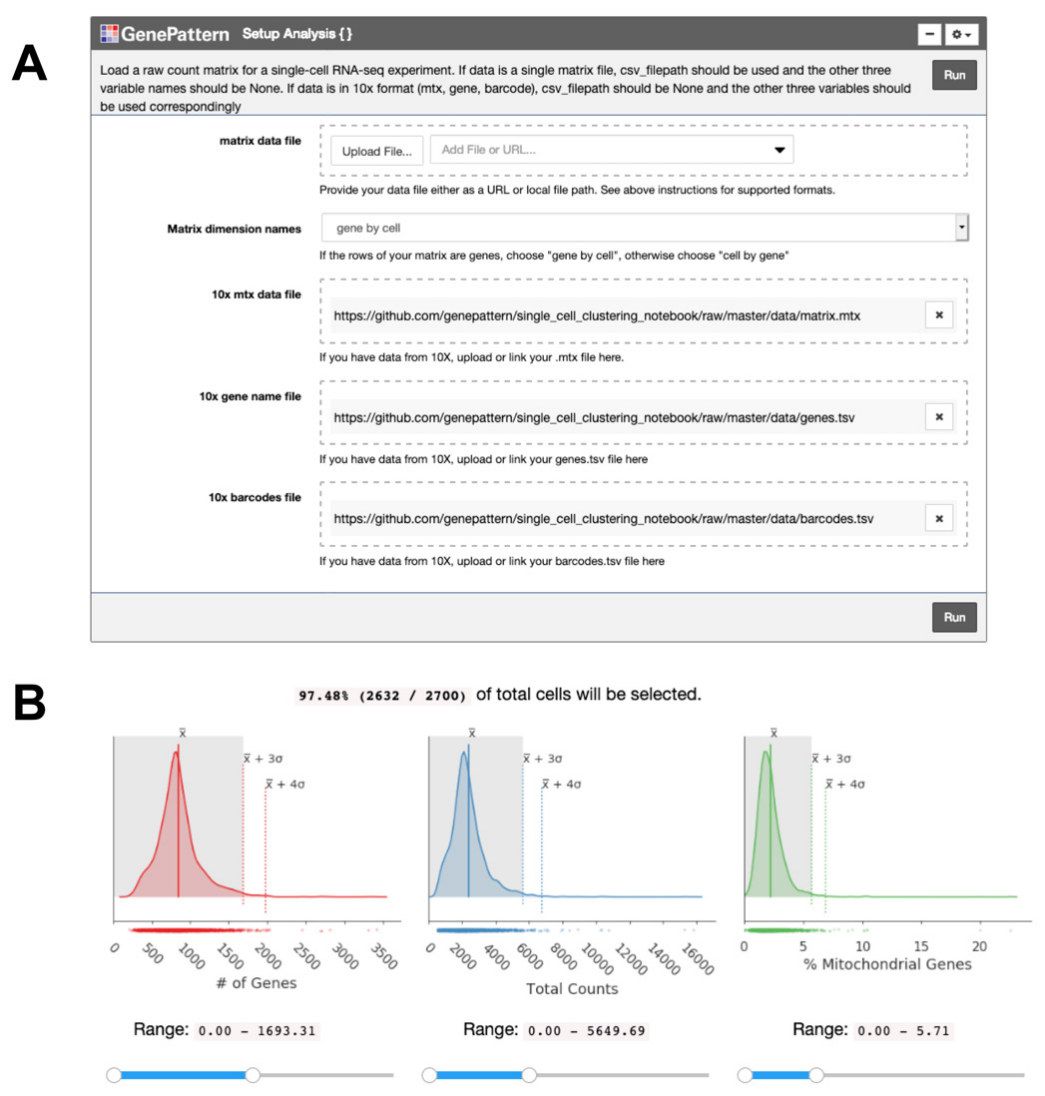
Cell quality metrics. ( **A**) The “Setup Analysis” function is presented using the GenePattern UI Builder. ( **B**) The quality metric distributions are shown as kernel density estimation fitted curves. The values of the mean, 3 standard deviations (SDs) above the mean and 4 SDs above the mean are indicated to help identify outlier cells with abnormally large metric values. Interactive sliders under each plot allow the user to see how many cells are included under a threshold.

Once the expression matrix is loaded into the notebook using a GenePattern cell ( Figure 1A), the notebook presents a series of plots to compare quality metrics across cells ( Figure 1B). There are 3 metrics including: the number of genes detected in each cell, the total counts in each cell, and, when available, the percentage of counts mapped to mitochondrial genes. A high percentage of mitochondrial genes indicates apoptotic or lysed cells. These disrupted cells tend to lose cytoplasmic RNA and retain RNA enclosed in the mitochondria. The user can interactively set thresholds to see how the number of cells below the threshold change ( Figure 1B). To use the mitochondrial gene filter, the user must supply their data with gene names in HGNC format with “MT-” prepended to each mitochondrial gene name.

### Preprocess counts

**Figure 2.**
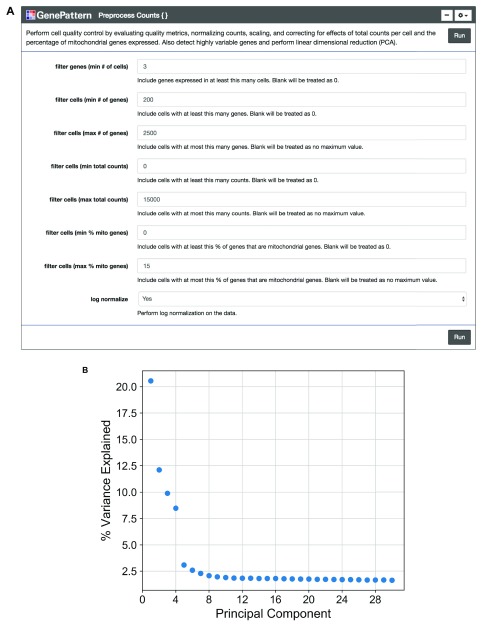
Preprocessing count data. ( **A**) The “Preprocess Counts” function is presented using the GenePattern UI Builder. Here the user specifies thresholds for filtering samples and for performing log normalization. ( **B**) A scatterplot showing the percent variance explained by each individual principal component.

We encourage the user to visually inspect their data across several parameters, using the quality metric plots provided prior to proceeding with further analysis. Furthermore, we enable the user to determine appropriate filtering thresholds for each of the metrics to exclude low quality cells and outliers by inputting thresholds in the GenePattern cell interface ( Figure 2A). We have also provided an option to filter for genes expressed in a minimum number of cells. All preprocessing steps follow the Seurat and Scanpy workflows. Counts are scaled to have the same total counts for each cell. Highly variable genes are identified for downstream analysis by selecting genes with a minimum mean expression and dispersion; where dispersion is calculated as the log of the mean to variance ratio. Counts are then log-transformed to reduce the distribution skew and bring it closer to a normal distribution. We also give users the option to remove sources of technical variation by performing linear regression on the total number of molecules detected and the percentage of reads mapped to mitochondrial genes. As there is debate in the field concerning the correctness of using regression on covariates such as percent mitochondrial reads ( Batson, 2018) we have made this step optional. Finally, the counts for highly variable genes in each cell are scaled to unit variance and a mean of zero. For clustering cells in the next step, dimensionality reduction is performed using principal component analysis (PCA) on highly variable genes. A plot showing the percent variance explained of each principal component is then displayed so the user may choose a reasonable number of principal components for use in clustering ( Figure 2B). We note that this notebook is a living, open source document and can be modified as the single cell community’s perspectives on best practices evolves.

### Cluster cells

**Figure 3.**
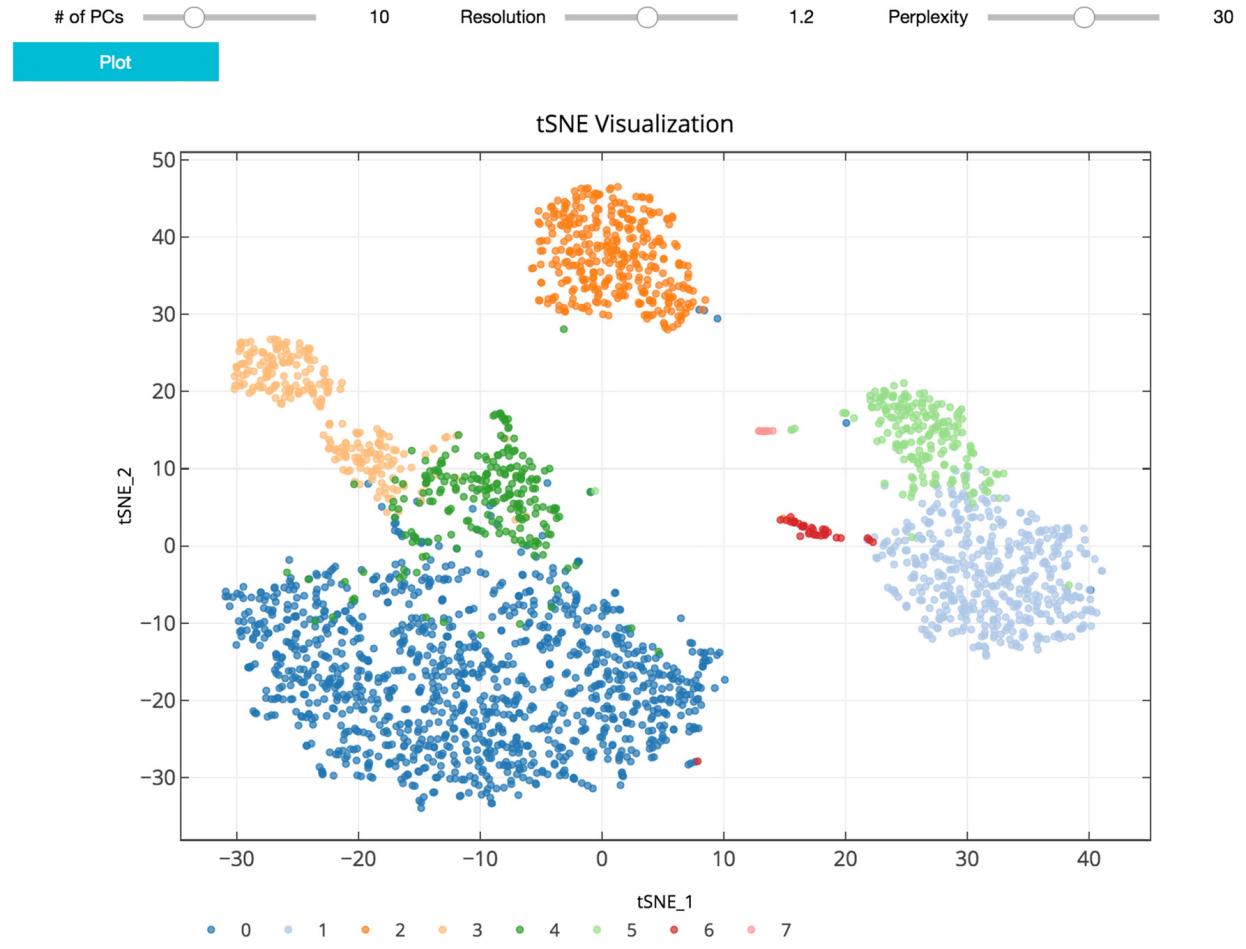
t-SNE plot visualizing cluster assignments of cells. The clustering parameters can be changed using the sliders and re-plotted with the “Plot” button. Cells are projected into t-SNE space, with the first two t-SNE components as the axes of the plot. Cluster assignments of cells are defined by Louvain clustering and denoted as distinct colors.

As suggested in Satija *et al*., 2015, and followed in the Seurat and Scanpy workflows, we cluster cells using a graph-based clustering approach. With the selected principal components as features, the cells are embedded in a K-nearest neighbor graph where cells are grouped using the Louvain community detection method ( Blondel *et al*., 2008). Then t-distributed stochastic neighbor embedding (t-SNE), a standard dimensionality reduction technique suited for visualizing high-dimensional data, is used to project and visualize the cells in the space of the first two t-SNE components ( Figure 3) ( Maaten & Hinton, 2008). Cells are represented as points colored by clustering assignment. Select parameters including the number of principal components, Louvain clustering resolution, and t-SNE perplexity are exposed for users to iteratively adjust the clustering results using the visualization for feedback ( Figure 3). Setting a higher resolution results in more and smaller clusters. The perplexity parameter loosely models the number of close neighbors each cell will have.

### Visualize cluster markers

**Figure 4.**
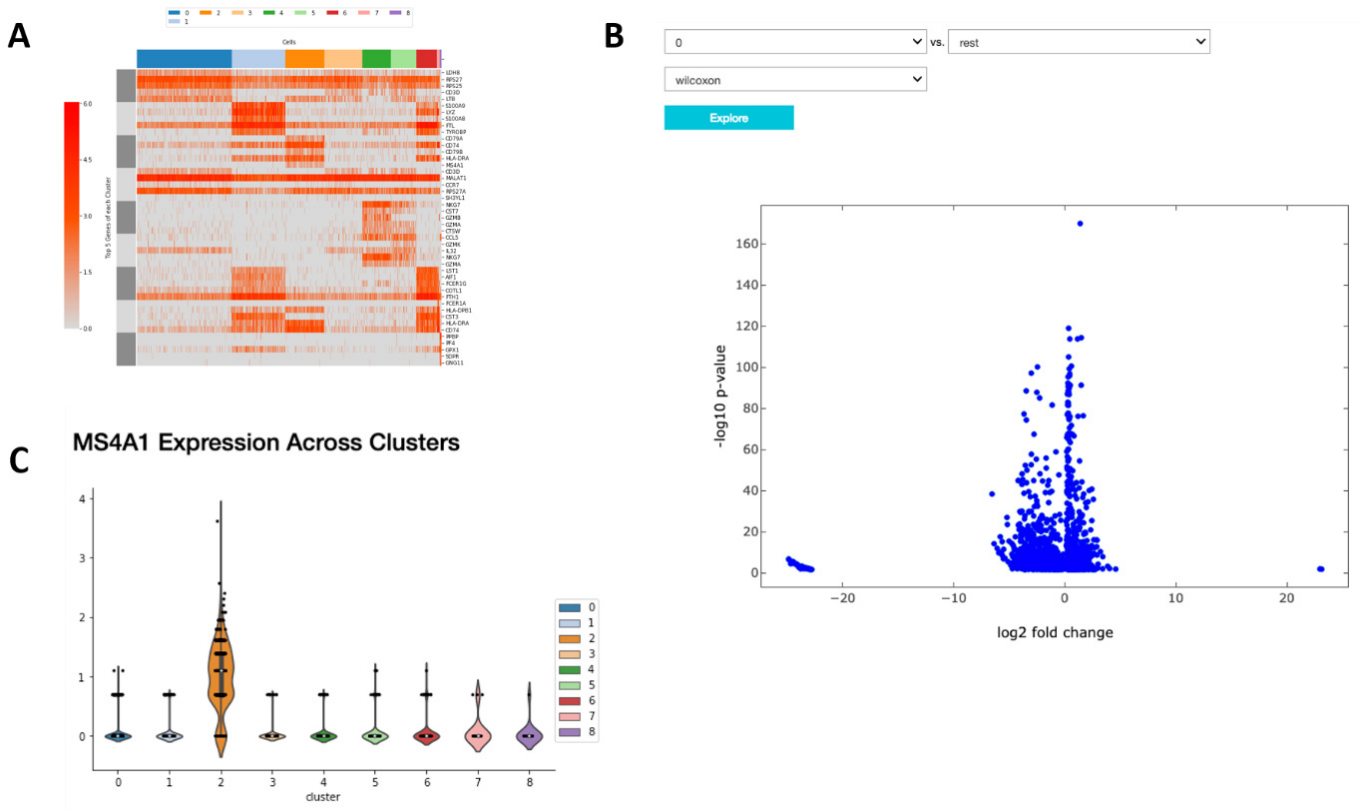
Relative expression of marker genes. ( **A**) A heatmap showing the expression of the top 10 differentially expressed markers of each cluster across all cells. ( **B**) A volcano plot illustrating the genes differentially expressed between two clusters or one cluster and the rest. ( **C**) A violin plot showing the expression of that gene in each cluster.

**Figure 5.**
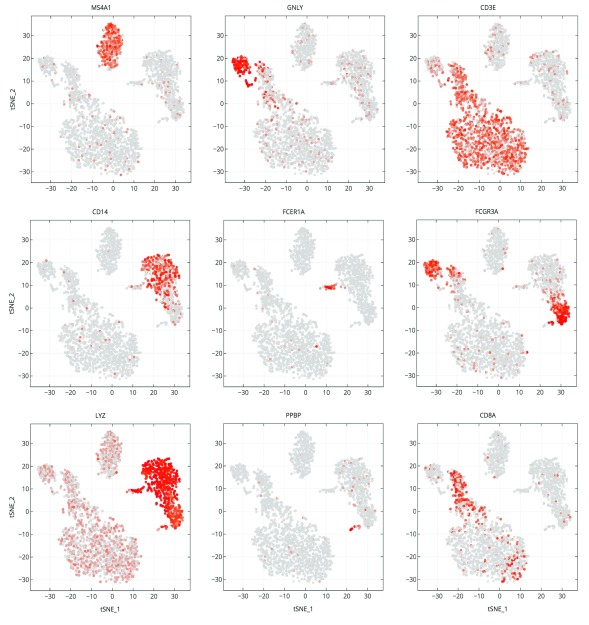
Marker gene expression projected on t-SNE plot. Cells are projected into t-SNE space, as in Figure 2, but are colored by the relative expression of a given gene instead of cluster assignment. Colors span a gradient from red (high expression) to grey (low expression). Genes shown here are indicative of known cell types; MS4A1: B cells, GNLY: NK cells, CD3E: T cells, CD14: CD14+ monocytes, FCER1A: dendritic cells, FCGR3A: FCGR3A+ monocytes, LYZ: CD14+ monocytes, PPBP: megakaryocytes, and CD8A: CD8 T cells.

The application of proper visualization tools is an important aid to interpret the complexity and depth of scRNA-seq data. We provide various visualizations within the notebook to explore differentially expressed genes, which can be used to identify specific cell types or highlight heterogeneous gene expression across clusters ( Figure 4A and B, Figure 5). There is also an interface to query for differentially expressed genes that are higher in one cluster compared to the rest ( Figure 4C). The Wilcoxon-Rank-Sum test statistic is used to rank genes by default. This test is performed in a one-versus-all setup for each of the clusters, providing unique markers for each individual cluster. We also include the option to perform pairwise cluster comparisons. Additional statistical information about each gene is provided in interactive plots, such as the log-fold change comparing the average expression of a gene in one cluster versus the average expression in all other cells, the percentage of cells within the cluster that express the gene, and the percentage of cells in other clusters that express the gene.

### Export analysis data

Data generated by the analysis can be exported in two ways. First the data can be exported as a set of CSV (comma separated values) files suited for further independent analysis and data sharing. We provide a description of the exported CSV files, which include the preprocessed expression matrix, cell annotations, dimensional reduction outputs, and gene rankings generated during the analysis. The data can also be exported as an H5AD file that can be re-imported into this notebook’s workflow, retaining the generated results. The parameters for each step in the analyses are automatically saved in the notebook once executed, ensuring the entire workflow is documented. Notably, the entire notebook can be shared with other users rather than exporting output files.

### Operation

To run this notebook, the user needs a GenePattern account or can create one on the GenePattern Notebook site. After logging in, the notebook can be found in the “Featured” section of the “Public Notebooks” page.

## Use case

An example notebook ( https://github.com/genepattern/single_cell_clustering_notebook) employs a scRNA-seq gene expression dataset for 2700 peripheral blood mononuclear cells (PBMCs) from a healthy donor as a demonstration of its use. We can recapitulate cell types identified using Seurat and Scanpy; the clusters can be characterized by visualizing the expression of canonical markers of these cell types on the 2D t-SNE projection plot. We also find that many of these markers are highly ranked when looking at significant differentially expressed genes between clusters ( Figure 4).

In Figure 4 we examine cluster markers to understand why some larger groups of cells are divided into sub clusters. For example, LYZ is overexpressed in a cloud of samples that clustering separates as two distinct clusters, 1 and 5. The LYZ gene encodes for human lysozyme, an antimicrobial agent associated with blood monocytes. Using the cluster comparison tool ( Figure 4B), we can see that cluster 1 exhibits high relative expression of CD14 while cluster 5 exhibits high relative expression of FCGR3A, also known as the CD16 receptor gene ( Figure 5). These two genes characterize two known subtypes of blood monocytes respectively; classical and non-classical monocytes.

## Conclusion

We encourage users to perform analyses on their own data using this notebook. We note that all the required libraries are already installed on the public GenePattern Notebook server at https://notebook.genepattern.org. This resource is freely available to the community and the analysis described in this notebook falls well within the per-account memory allocations (see the Scanpy authors’ benchmarking in Wolf *et al*., 2018; Eulenberg *et al*., 2017a; Eulenberg *et al.*, 2017b). To analyze larger datasets that exceed the per-user memory allocation on the public notebook server, users should deploy the open source GenePattern Notebook server using their own computational resources as described in Reich *et al*., 2017. The GenePattern Notebook server is available as the genepattern-notebook package through the pip ( https://pypi.org/project/genepattern-notebook/) or conda ( https://anaconda.org/genepattern/genepattern-notebook) package managers, or as a Docker image ( https://hub.docker.com/r/genepattern/genepattern-notebook).

As single-cell RNA-seq continues to grow in popularity, this GenePattern Notebook will provide an accessible and reproducible way to preprocess the data and perform clustering analysis without having to interact with any code. We plan to continually review the notebook as single-cell RNA-seq protocols evolve to be even more high-throughput and as algorithms adapt to accommodate growing amounts of single-cell data. For example, future notebook releases may include quality control methods such as doublet detection ( McGinnis *et al*., 2018) as well as visualization methods such as UMAP ( Becht *et al*., 2019), which is growing in popularity in the single cell community. We also encourage advanced users to copy the notebook, add new approaches or features, and publish them as a community notebook in the GenePattern Notebook repository. As the GenePattern Notebook user interface gains more features, the notebook will also be able to take advantage of these features. Future notebooks such as those for multi-experiment aggregation (multiple sequencing runs) and pseudotime analysis are being considered to grow a compendium of single-cell sequencing analysis notebooks.

## Software and data availability

GenePattern Notebook Web site https://genepattern-notebook.org. GenePattern Notebook repository and workspace: https://notebook.genepattern.org/.

GenePattern Notebook source code is available from: https://github.com/genepattern/seurat_python_notebook.

GenePattern Notebook and all its dependencies are available as a Docker image: https://hub.docker.com/r/genepattern/genepattern-notebook

Archived source code as at time of publication: https://doi.org/10.5281/zenodo.2584417 ( Mah, 2019)

License: BSD 3-Clause

The 3k PBMCs from a Healthy Donor dataset is publicly available via the 10X Genomics website after user registration: https://support.10xgenomics.com/single-cell-gene-expression/datasets/1.1.0/pbmc3k.
