# Supplementary material for: An accessible, interactive GenePattern Notebook for analysis and exploration of single-cell transcriptomic data
Source: F1000Res. 2019 May 29;7:1306. Originally published 2018 Aug 16. [Version 2] doi: 10.12688/f1000research.15830.2 (PMC6611141; doi:10.12688/f1000research.15830.2)
Supplement: Peer review history

# Peer review: Reviewer response for version 2

**DOI:** 10.5256/f1000research.20652.r49213

**Published:** 2019-07-01

**Authors:** Timothy Tickle

**Affiliations:** 1 The Broad Institute of MIT and Harvard, Cambridge, MA, USA

Thank you to the authors of the paper for their time and effort in addressing comments in the previous revision. In the previous review, the following major comments were requested to be addressed: 1) extending the differential expression to more than two labels, 2) a suggestion of using dot plots over heatmaps, 3) a request for benchmarking execution time, and 4) a clarification of costs.

 Admittedly, the first two points could be interpreted as extending and not delivering the specific tutorial that is being replicated. In this light, Seurat tutorials are a de facto standard and making them available "as is" in this notebook environment adds great value to the community. I look forward to the community extending these notebooks with additional functionality but seeing how this works is not needed for the indexing of this manuscript. Execution time for each step is now given in the notebook for major steps. Costs are clarified as part of the conclusion of the manuscript. These corrections and actions address the requested points from the previous review.

I confirm that I have read this submission and believe that I have an appropriate level of expertise to confirm that it is of an acceptable scientific standard.

# Peer review: Reviewer response for version 2

**DOI:** 10.5256/f1000research.20652.r49214

**Published:** 2019-06-11

**Authors:** Joshua Batson

**Affiliations:** 1 CZ Biohub, San Francisco Bay Area, CA, USA

The revisions to the visualizations and to file I/O are appreciated. The implementation of the method put forward in Seurat's first PBMC tutorial is sound. As the authors state, ongoing research in single-cell will necessitate that this be a living document in the same way that the major software packages are.

I confirm that I have read this submission and believe that I have an appropriate level of expertise to confirm that it is of an acceptable scientific standard.

# Peer review: Reviewer response for version 1

**DOI:** 10.5256/f1000research.17278.r40612

**Published:** 2018-11-15

**Authors:** Joshua Batson

**Affiliations:** 1 CZ Biohub, San Francisco Bay Area, CA, USA

This manuscript describes a GenePattern Notebook implementing a standard analysis pipeline for single-cell RNA sequencing data. GenePattern notebooks allow a user to access python libraries for data analysis through a simple GUI--dropdown menus, text fields, and sliders are used to provide inputs to python functions that get run under the hood. This allows a user familiar with web interfaces to run a program without having to understand syntax.

 In this case, that program is Scanpy, a package for scRNA-seq analysis. The order of the steps and the supporting guidelines for parameter choice and data exploration are taken from the Seurat PBMC3k tutorial.

 Ideally, a naive user could upload their gene counts (say the output of CellRanger by 10X), tune just a few parameters according to the guidelines in the notebook, and find meaningful clusters in their data. Such an accessible analysis with guard-rails would be useful--had I been aware of the GenePattern system when organizing data analysis for the Tabula Muris project, I would certainly have used it, and so avoided many problems with people deleting code they shouldn't have, not changing file names to match their local directory structure, etc.

 There are some significant usability issues with the notebook as it stands that will need to be remedied for it to be useful to a broad audience. Since people who cannot program will be unable to make even small changes to the workflow, it is important that the pipeline implemented here by scientifically sound, robust to different input formats and scales, and feature-complete.

- Data I/O. I attempted to use the web-hosted version of the notebook to upload a large csv (80 MB). After nothing happened, I switched to a smaller CSV. Since it was in gene x cell format instead of cell x gene, the analysis couldn't proceed. Finally I tried a 10X .mtx file, but as there was no way to upload the corresponding barcodes file, that didn't work. Finally I used an h5ad file from a previous scanpy analysis. That won't be possible for the typical user. (Given slow upload times, actually, you may want to accept zipped csvs as well.)

The author should make it possible to upload 10X files properly. They should also allow for the data to be gene x cell or cell x gene, by giving the user a chance to transpose the matrix if necessary.

- Interactive sliders are nice, much better than setting cutoffs by number, seeing how values change, and iterating. For the cutoffs on nGene and nCounts, the sliders are not aligned with the plot and lack numerical axes or numbers for current values. These should be displayed directly under the plots so that one can slide them to align with suggested cutoffs at various values of sigma, and should also display the numerical value currently selected.

 (Also, the suggestion that 3\sigma is an appropriate cutoff is based on the assumption of a normal distribution, which does not hold for this data, especially for 10X.)

- The notebook bakes in certain analysis choices, such as regressing out % mito, which are not statistically sound. For the problems with regressing out, see this [blog post](http://ds.czbiohub.org/blog/Regression-Hazards/). I suggest that such "corrections" be removed. For a simple, sound analysis, see the workflow and language we used for Tabula Muris [Annotation Vignette](https://github.com/czbiohub/tabula-muris/blob/master/00_data_ingest/02_tissue_analysis_rmd/Organ_Annotation_Vignette.Rmd)
- The notebook also includes some assumptions about the reference used. In particular, it assumes for % mito that genes be formatted in a certain case.
- Users will likely have metadata they want to visualize, such as sample, batch, sex, stimulated vs unstimulated, etc. They should be able to upload a csv with that data, and visualize it on the tSNE plots. This is important for interpretation of the results.
- The tSNE tab for visualizing gene expression did not load when clicked on.
- "Conversely, reads for multiple cells may be captured together, artificially inflating the number of reads for a single cell. " Doublet detection is indeed a tricky problem. There are [methods](https://www.biorxiv.org/content/early/2018/07/19/352484) to address it, but this notebook does not implement them.
- Depending on the size of the data, each step may take seconds to hours. For a naive user, they may not know how long to wait and at some point anyone would give up. It would be very useful if the author did some calibration for each step (running sample datasets of various sizes) so that an estimated time to completion could be displayed.

Final remarks:

 Single-cell sequencing analysis is evolving, and it is essential that we get the most sound methods in the hands of practitioners. Anchoring this notebook to Scanpy is great, since that library is actively being developed. I recommend that the author regularly update this template with methods as they become available in that library. For example, t-tests and the wilcox have problems with log-normalized data (they fail to be consistent when cells are sampled to different depths). I recommend the t-test_overestim_var from Scanpy for something fast and logreg for something more accurate, but all the options should be made available and the defaults from Scanpy should be the defaults for you.

 This will need to be a living document to be useful. If it is a repository of best practices for simple single-cell analysis, then it may serve as a way in to single for people who don't yet know how to program.

I confirm that I have read this submission and believe that I have an appropriate level of expertise to confirm that it is of an acceptable scientific standard, however I have significant reservations, as outlined above.

# Response

**Published:** 2018-12-17

**Authors:** Jill Mesirov

**Affiliations:** Department of Medicine, University of California, San Diego, USA

We appreciate your comprehensive comments and suggestions for improvement and enhancement of the notebook and are working to incorporate them as well as revise the paper. When that work is finished we will provide a more detailed response to review.

# Response

**Published:** 2019-04-03

**Authors:** Clarence Mah

**Affiliations:** UC San Diego, USA

*1. The current manuscript focuses on user experience through a standardized analysis pattern, this is done well by the publication. The majority of the analysis and data visualization is modeled after current trends but two areas of improvement exist. The use of a Wilcoxon-Rank-Sum test to test one label verse all other labels is useful to researchers and is something offered through current analysis packages. There has been work that has improved the way differential expression is performed, SCDE and MAST are packages that allow more complex models or comparisons that go beyond two labels. Of those, at least MAST has shown to be performant and is now available as an option in Seurat. It is rare one will only find two clusters of cells in a single cell transcriptomics study, the extension to more labels should be included.*

 We agree with the reviewer that other analyses, for example more complex differential expression, can be performed. However, writing suitable Python wrappers for those is beyond the scope of this article and notebook. As Scanpy is constantly updated to reflect these current best practices to analyze single cell RNA-Seq data, this notebook can be updated as well.

*2. Heatmaps were used in Figure 4a. Although useful when one wants to see the actual data and scalable to an extremely small subsection of genes (here 10), often single cell transcriptomics data is too sparse to fully appreciate patterns when presented in heatmaps (as measurements, when not using summaries) and the numbers of observations in these studies can go to more than a million; making this visualization not scalable. Dot plots should be the first plot offered in these use cases with the ability to go to heatmaps to see the actual data if needed.*

 We first note that, due to software compatibility issues with visualization widgets, we have redesigned the presentation of Figure 4 in the manuscript and the visualization in Step 4 of the notebook. However, we have chosen, in this case, to continue displaying the heatmap first. We agree with the reviewer that heatmaps for large, sparse data are useful mostly for summarizing, and that is indeed the reason we provide this heatmap. Users can identify the top *n* markers of each cluster in the heatmap (now Step 4A) and then explore each marker in more detail using the tSNE and violin plots in Step 4B.

*3. Standardization and approachable user experiences are big wins for our community, but this has to be coupled with an underlying methodology that is scalable to the sizes of data we see and expect to see the near future. Data sets of over a million already exist, how does this solution scale to data in the thousands, tens of thousands, and so on. It is essential the manuscript include benchmarking so users can understand if working with their data set in this environment is possible.*

 We have included benchmarking relevant to the Seurat PBMC tutorial dataset before each step in the notebook. Additionally, the Scanpy development team has benchmarked their code (which forms the core of the analysis performed by this notebook) on both the same Seurat tutorial dataset we use in the notebook and a large dataset of one million cells. We have cited these benchmarks in the manuscript (see response to Reviewer 1). We note that the GenePattern Notebook uses a client-server architecture with most processing done by the server. Most datasets on the order of thousands of cells can likely be processed using the free public GenePattern Notebook server at https://notebook.genepattern.org. For larger datasets, the open source GenePattern Notebook server can be deployed on any appropriate computational resource, for example, an Amazon AWS instance or an institutional supercomputer allocation. We have added text to the manuscript noting this – see point 4 below.

*4. Please list if there are any costs (or that there are no costs if that is, in fact, true) with running analysis in the notebooks. Must one always download the notebooks and run them on their own systems or is the running of analysis hosted?*

 We have added the following text to the conclusion section of the manuscript to address this concern:

 We encourage users to perform analyses on their own data using this notebook. We note that all the required libraries are already installed on the public GenePattern Notebook server at https://notebook.genepattern.org. This resource is freely available to the community and the analysis described in this notebook falls well within the per-account memory allocations (see the Scanpy authors’ benchmarking in Wolf et al., 2018, Eulenberg et al., 2017, Eulenberg et al. 2, 2017). To analyze larger datasets that exceed the per-user memory allocation on the public notebook server, users should deploy the open source GenePattern Notebook server using their own computational resources as described in Reich et al., 2017. The GenePattern Notebook server is available as the genepattern-notebook package through the pip ( https://pypi.org/project/genepattern-notebook/) or conda ( https://anaconda.org/genepattern/genepattern-notebook) package managers, or as a Docker image ( https://hub.docker.com/r/genepattern/genepattern-notebook).

*A response to following minor comments are of interest to the reviewer.*

*1. It is interesting that Jupyter Notebooks are leveraged to target an audience that can not program given Jupyter notebooks are a common environment for developers. That being said the interfaces given to the users do seem to be appropriate user experiences for those who prefer working through UIs. Does this work also include the ability for someone to edit and update the code of the GenePattern Notebook if they are a developer? Such a functionality would extend the usability of the notebooks by supporting an additional type of user.*

 Yes, GenePattern Notebooks allow users who are comfortable programming to modify the underlying code or add their own. Users access public GenePattern notebooks by saving a copy of the notebook to their own accounts. In addition to executing the analysis with the notebook copy, users can modify their copy, share it with other users, and publish modified versions to a notebook repository. Please refer to the article describing GenePattern Notebook for further details ( https://doi.org/10.1016/j.cels.2017.07.003) or to the section on Programmatic Features of the GenePattern Notebook website ( http://genepattern-notebook.org/programmatic/).

*2. In Figure 1 kernel density estimations with a data plotted below the density are used instead of violin plots with over-plotted data. Although I appreciate the same information is being presented in both plots (hence this being a minor comment), it would be helpful to use violin plots instead of the current plots. First, violin plots are de facto (and the GenePattern plot does not add information) and secondly, outliers are given more presence in violin plots (data is plotted directly on the plot and the tail of the plot to higher values is not hidden by an axis). This is important given outlier are explicitly the focus of figure 1.*

 In Figure 1, cells are displayed under the density plots (in the form of dots) with outliers clearly visible. We believe this already implemented functionality achieves the effect of highlighting the outliers in the distribution as requested by the reviewer.

# Peer review: Reviewer response for version 1

**DOI:** 10.5256/f1000research.17278.r37288

**Published:** 2018-09-17

**Authors:** Timothy Tickle

**Affiliations:** 1 The Broad Institute of MIT and Harvard, Cambridge, MA, USA

“An accessible, interactive GenePattern Notebook for analysis and exploration of single-cell transcriptomic data” by Mah et al announces GenePattern NoteBooks to provide an interactive, easy-to-use interface for data analysis and exploration of single cell transcriptomics data.

 GenePattern is an important body of software that supports an active user base. Growing resources for single cell transcriptomics will continue to enable that community and extend GenePattern to additional users. Jupyter notebooks are an excellent tool for development and reproducibility of scripts, it is interesting to see it being leveraged as a space to provide user interfaces for single cell transcriptomics data science. The selection of analysis was well selected, using a tutorial from Seurat, a leading environment for single cell transcriptomics analysis who have historically provided a collection of high-quality tutorials. Steps for analysis leveraged from that tutorial seem reasonable and visualizations (excluding one comment below) seem to match what you would see in publications.

 The following major comments should be addressed.

1. The current manuscript focuses on user experience through a standardized analysis pattern, this is done well by the publication. The majority of the analysis and data visualization is modeled after current trends but two areas of improvement exist. The use of a Wilcoxon-Rank-Sum test to test one label verse all other labels is useful to researchers and is something offered through current analysis packages. There has been work that has improved the way differential expression is performed, SCDE and MAST are packages that allow more complex models or comparisons that go beyond two labels. Of those, at least MAST has shown to be performant and is now available as an option in Seurat. It is rare one will only find two clusters of cells in a single cell transcriptomics study, the extension to more labels should be included.
2. Heatmaps were used in Figure 4a. Although useful when one wants to see the actual data and scalable to an extremely small subsection of genes (here 10), often single cell transcriptomics data is too sparse to fully appreciate patterns when presented in heatmaps (as measurements, when not using summaries) and the numbers of observations in these studies can go to more than a million; making this visualization not scalable. Dot plots should be the first plot offered in these use cases with the ability to go to heatmaps to see the actual data if needed.
3. Standardization and approachable user experiences are big wins for our community, but this has to be coupled with an underlying methodology that is scalable to the sizes of data we see and expect to see the near future. Data sets of over a million already exist, how does this solution scale to data in the thousands, tens of thousands, and so on. It is essential the manuscript include benchmarking so users can understand if working with their data set in this environment is possible.
4. Please list if there are any costs (or that there are no costs if that is, in fact, true) with running analysis in the notebooks. Must one always download the notebooks and run them on their own systems or is the running of analysis hosted?

A response to following minor comments are of interest to the reviewer.

1. It is interesting that Jupyter Notebooks are leveraged to target an audience that can not program given Jupyter notebooks are a common environment for developers. That being said the interfaces given to the users do seem to be appropriate user experiences for those who prefer working through UIs. Does this work also include the ability for someone to edit and update the code of the GenePattern Notebook if they are a developer? Such a functionality would extend the usability of the notebooks by supporting an additional type of user.
2. In Figure 1 kernel density estimations with a data plotted below the density are used instead of violin plots with overplotted data. Although I appreciate the same information is being presented in both plots (hence this being a minor comment), it would be helpful to use violin plots instead of the current plots. First, violin plots are de facto (and the GenePattern plot does not add information) and secondly, outliers are given more presence in violin plots (data is plotted directly on the plot and the tail of the plot to higher values is not hidden by an axis). This is important given outlier are explicitly the focus of figure 1.

I confirm that I have read this submission and believe that I have an appropriate level of expertise to confirm that it is of an acceptable scientific standard, however I have significant reservations, as outlined above.

# Response

**Published:** 2019-04-03

**Authors:** Clarence Mah

**Affiliations:** UC San Diego, USA

*1. Data I/O. I attempted to use the web-hosted version of the notebook to upload a large csv (80 MB). After nothing happened, I switched to a smaller CSV. Since it was in gene x cell format instead of cell x gene, the analysis couldn't proceed. Finally I tried a 10X .mtx file, but as there was no way to upload the corresponding barcodes file, that didn't work. Finally I used an h5ad file from a previous scanpy analysis. That won't be possible for the typical user. (Given slow upload times, actually, you may want to accept zipped csvs as well.)*

*The author should make it possible to upload 10X files properly. They should also allow for the data to be gene x cell or cell x gene, by giving the user a chance to transpose the matrix if necessary.*

We have addressed the lack of flexibility in regards to input files and mention that in the text.

Users can now upload or link to their own 10X files (matrix, genes, and barcodes) or upload a single matrix file and specify whether it contains “gene by cell” or “cell by gene” data. Any of these files may be zipped as well. The new text reads as follows:

“The workflow begins with an expression data matrix already derived from alignment of reads and quantification of RNA transcripts. Users may upload a single expression file and specify whether the rows represent genes and the columns represent cells or vice-versa. Text files from read count quantification tools like HTSeq ( Anders et al. 2015) and Kallisto ( Bray et al. 2016) are supported as input. Additionally, this notebook supports the three-file 10X output format, allowing users to upload the matrix, genes, and barcodes files. Any of those inputs can also be provided as .zip files.”

We thank the reviewer for providing additional files for us to use in testing and we have confirmed that the notebook will load even the larger 80MB file.

*2. Interactive sliders are nice, much better than setting cutoffs by number, seeing how values change, and iterating. For the cutoffs on nGene and nCounts, the sliders are not aligned with the plot and lack numerical axes or numbers for current values. These should be displayed directly under the plots so that one can slide them to align with suggested cutoffs at various values of sigma, and should also display the numerical value currently selected.*

*(Also, the suggestion that 3 is an appropriate cutoff is based on the assumption of a normal distribution, which does not hold for this data, especially for 10X.)*

We have adjusted the alignment of plot elements such that the sliders are now more closely aligned with the boundaries of their respective KDE plots. We have left the graphical markers indicating the third and fourth standard deviation on the plots for the purpose of describing the distribution, but we have removed the suggestion that those values may make good cutoffs.

*3. The notebook bakes in certain analysis choices, such as regressing out % mito, which are not statistically sound. For the problems with regressing out, see this [blog post](* *http://ds.czbiohub.org/blog/Regression-Hazards/* *). I suggest that such "corrections" be removed. For a simple, sound analysis, see the workflow and language we used for Tabula Muris [Annotation Vignette](* *https://github.com/czbiohub/tabula-muris/blob/master/00_data_ingest/02_tissue_analysis_rmd/Organ_Annotation_Vignette.Rmd* *)*

We appreciate the reviewer's word of caution with regards to regressing out %mito and the links to some relevant literature. We included some of these analysis choices as we are explicitly following the Seurat tutorial, and that is a data preprocessing step in the tutorial. However, we added a note of caution to the text of the manuscript and the GenePattern notebook with regards to the possible hazards of regression in the single cell RNA-seq context while noting the alternatives provided, and we have made the regression step optional. The text now reads as follows:

We also give users the option to use remove sources of technical variation by performing linear regression on the total number of molecules detected and the percentage of reads mapped to mitochondrial genes. As there is debate in the field concerning the correctness of using regression on covariates such as percent mitochondrial reads (Batson 2018) we have made this step optional.

*4. The notebook also includes some assumptions about the reference used. In particular, it assumes for % mito that genes be formatted in a certain case.*

For the purposes of reproducing the Seurat tutorial, we kept the convention of assuming that mitochondrial gene names begin with “MT-”. This “hardcoding” could be avoided by asking users to supply a list of mitochondrial genes corresponding to their data’s gene ID system, but we feel this might over-complicate the input step and impose a burden on users, especially since, as the reviewer observes, the percent mitochondrial genes regression is of questionable statistical utility in the general case. We have emphasized in the manuscript that gene names must follow this specific convention in order to use this regression. Because of this requirement as well as the statistical issues the reviewer notes above, we have made this regression step optional. Users who either do not wish to use regression or do not have gene names in the required format can now skip this step.

*5. Users will likely have metadata they want to visualize, such as sample, batch, sex, stimulated vs unstimulated, etc. They should be able to upload a csv with that data, and visualize it on the tSNE plots. This is important for interpretation of the results.*

We agree with the reviewer that this is an important step in single cell RNA-seq workflows. However, an appropriate implementation of visualization of metadata is a feature that we believe would require a separate notebook and is out of scope of the goal of this particular notebook. Thus, we leave this for future work.

*6. The tSNE tab for visualizing gene expression did not load when clicked on.*

We have identified the source of this error as an incompatibility between GenePattern Notebooks and certain Jupyter Widgets (ipywidgets) that arose after the initial release of this notebook. We are addressing this problem; however, in order to avoid further delays we have redesigned the visualization without using the incompatible widgets. Correspondingly, Figure 4 in the manuscript has been updated to reflect this change.

*7. Conversely, reads for multiple cells may be captured together, artificially inflating the number of reads for a single cell. " Doublet detection is indeed a tricky problem. There are [methods](https://www.biorxiv.org/content/early/2018/07/19/352484) to address it, but this notebook does not implement them.*

We appreciate the importance of considering problems such as doublet detection. The Seurat PBMC tutorial does not include a doublet detection step and Scanpy does not currently implement methods for handling doublets. However, we do mention the importance of this issue in the text and point to a reference, and that we hope to address this issue in future work. The relevant text reads as follows:

For example, future notebook releases may include quality control methods such as doublet detection ( McGinnis *et al.,* 2018) as well as visualization methods such as UMAP ( Becht *et al.,* 2019), which is growing in popularity in the single cell community.

*8. Depending on the size of the data, each step may take seconds to hours. For a naive user, they may not know how long to wait and at some point anyone would give up. It would be very useful if the author did some calibration for each step (running sample datasets of various sizes) so that an estimated time to completion could be displayed.*

We have annotated each step in the notebook with benchmarking for the Seurat PBMC dataset. For each step, we list the default amount for the limiting factors (genes, principal components, etc.) and the execution time we estimate for that step based on those values. For example, “For 2,600 cells and 10 principal components, this step will run in approximately 60 to 90 seconds”. Additionally, the Scanpy developers have benchmarked their code both on the same Seurat PBMC dataset we use in this notebook and on an large dataset of one million cells. We have cited these benchmarks in the manuscript in the first paragraph of the conclusions.

*Final remarks:*

*Single-cell sequencing analysis is evolving, and it is essential that we get the most sound methods in the hands of practitioners. Anchoring this notebook to Scanpy is great, since that library is actively being developed. I recommend that the author regularly update this template with methods as they become available in that library. For example, t-tests and the wilcox have problems with log-normalized data (they fail to be consistent when cells are sampled to different depths). I recommend the t-test_overestim_var from Scanpy for something fast and logreg for something more accurate, but all the options should be made available and the defaults from Scanpy should be the defaults for you.*

*This will need to be a living document to be useful. If it is a repository of best practices for simple single-cell analysis, then it may serve as a way in to single for people who don't yet know how to program.*

We agree with the reviewer that keeping this notebook in line with evolving best practices is essential. We also agree that Scanpy’s default parameters are likely robust in the general case, however, in keeping with the objective of following the Seurat PBMC tutorial, default settings for parameters in this notebook were chosen to reproduce the results from that tutorial. We have included documentation at each step in the notebook to introduce users to the purpose and nuance of different parameters and empower them to make the best choices for their own data.
